# Cafeteria diet impairs expression of sensory-specific satiety and stimulus-outcome learning

**DOI:** 10.3389/fpsyg.2014.00852

**Published:** 2014-08-27

**Authors:** Amy C. Reichelt, Margaret J. Morris, R. F. Westbrook

**Affiliations:** ^1^School of Medical Sciences, The University of New South WalesSydney, NSW, Australia; ^2^School of Psychology, The University of New South WalesSydney, NSW, Australia

**Keywords:** obesity, sensory-specific satiety, devaluation, incentive value, Pavlovian conditioning

## Abstract

A range of animal and human data demonstrates that excessive consumption of palatable food leads to neuroadaptive responses in brain circuits underlying reward. Unrestrained consumption of palatable food has been shown to increase the reinforcing value of food and weaken inhibitory control; however, whether it impacts upon the sensory representations of palatable solutions has not been formally tested. These experiments sought to determine whether exposure to a cafeteria diet consisting of palatable high fat foods impacts upon the ability of rats to learn about food-associated cues and the sensory properties of ingested foods. We found that rats fed a cafeteria diet for 2 weeks were impaired in the control of Pavlovian responding in accordance to the incentive value of palatable outcomes associated with auditory cues following devaluation by sensory-specific satiety. Sensory-specific satiety is one mechanism by which a diet containing different foods increases ingestion relative to one lacking variety. Hence, choosing to consume greater quantities of a range of foods may contribute to the current prevalence of obesity. We observed that rats fed a cafeteria diet for 2 weeks showed impaired sensory-specific satiety following consumption of a high calorie solution. The deficit in expression of sensory-specific satiety was also present 1 week following the withdrawal of cafeteria foods. Thus, exposure to obesogenic diets may impact upon neurocircuitry involved in motivated control of behavior.

## INTRODUCTION

Access to highly palatable and calorically rich foods is a major contributing factor to the increasing rates of obesity worldwide (Caballero, 2007). Eating is essential for survival and is underpinned by the fundamental physiological need to consume energy. However, our basic requirements for nutrients and energy to maintain physiological homeostasis are often exceeded by an abundant source of readily available and convenient sources of foods and drinks. Consumption beyond basic homeostatic needs, purely based on the rewarding properties of palatable foods, is proposed to be a central contributor to the current worldwide obesity epidemic (Berthoud, 2004).

A range of animal and human data demonstrates that excessive consumption of palatable food leads to changes in the sensitivity of brain reward circuitries. These reward pathways are highly conserved across species and have been associated with altered responsiveness to reward (e.g., food) in obesity. Studies have demonstrated diminished responsiveness to perform food motivated behaviors and rewarding intracranial self-stimulation in obese rats (Volkow and Wise, 2005; la Fleur et al., 2007; Pickering et al., 2009; Johnson and Kenny, 2010) and reduced sensitivity to reward (measured by ratings of motivation and pleasure derived from engaging in rewarding behaviors) in obese humans (Davis et al., 2004).

Reward-based feeding, or eating for pleasure, can be prompted by learning that certain highly palatable foods are associated with discrete cues. Studies using functional brain imaging in obese subjects show that palatable foods and food-associated cues increase activity in cortical regions associated with motivational control and reward-based feeding including the orbitofrontal cortex (OFC), insula, amygdala, hypothalamus, striatum, and midbrain regions including the ventral tegmental area (VTA; Wang et al., 2001; Stice et al., 2008; Martin et al., 2010).

It has been proposed that sensitivity to cues predictive of food reward is increased in obesity (Stice et al., 2008), and may modulate the associative properties of food-related cues, evoking cravings for particular foods, triggering over-consumption (Meule et al., 2012; Jastreboff et al., 2013; Meule et al., 2014). Reducing the incentive value of a particular food associated with an operant response or a conditioned stimulus (CS) by lithium-induced devaluation, or pre-feeding to satiety decreases performance of particular responses (Dickinson et al., 1996; Balleine and Dickinson, 1998; Reichelt et al., 2011, 2013). Recently, rats ingesting a sucrose solution or a high fat/high sugar solution were shown to demonstrate impairments in outcome devaluation in an operant setting (Kendig et al., 2013; Furlong et al., 2014), indicating that consumption of high-energy foods can induce differences in reward-oriented instrumental behavior. This value driven control of responding has also been observed in a Pavlovian setting, whereby rats will reduce food seeking (goal-tracking or magazine-approach) behaviors associated with presentation of a CS whose associated unconditioned stimulus (US) has been separately devalued (Pickens et al., 2003, 2005; Ostlund and Balleine, 2007; Johnson et al., 2009; Lelos et al., 2011). These results suggest that the motivational value of a palatable outcome can control the performance of food seeking behaviors and if these associations are maladaptive, cues may promote responding regardless of whether the food is valued, so evoking over-eating. Another notion is that obesity may enhance resistance to satiation (Morgan, 1974; Capaldi et al., 1981), whereby a sated animal will continue to perform an instrumental response to gain food reward even when the incentive value of food is low. This concept bears many similarities to habitual responding, whereby a well-practiced behavior can be evoked through the presence of a stimulus alone (Dickinson et al., 1995; Killcross and Coutureau, 2003).

In addition to food-associated cues promoting consumption, the variety of foods in diets have also been shown to influence consumption. Animal and human studies show that food consumption increases when there is more variety in a meal or diet and that greater dietary variety is associated with increased body weight and adiposity. The presentation of a wide range of foods evokes over-eating, known as the “buffet effect” (Rolls et al., 1981; Rolls, 1984). This overeating plays an important role in food choice and meal termination, and may constitute one of the mechanisms that contributes to obesity. This enhancement of food consumption when presented with a variety of available foods may have an evolutionary advantage, potentially to prevent nutritional deficiencies (Rolls, 1981). The converse of the variety effect is the depressed consumption when the diet is unchanged. This depression is likely due to sensory-specific satiety, which has been defined as the decrease in the hedonic pleasantness of a food after it is eaten (Snoek et al., 2004). This decrease in the palatability of a consumed food shifts preference toward other foods, resulting in their consumption (Rolls, 1981). Following satiation on one food mice, rats, and primates also choose to eat an alternative food (Rolls et al., 1989; Dickinson et al., 1996; Balleine and Dickinson, 1998; Ahn and Phillips, 1999; Reichelt et al., 2011, 2013; Ahn and Phillips, 2012).

Animals rapidly gain weight when provided with a variety of foods (cafeteria diet) compared to a diet of just one food (Rolls et al., 1981) suggesting that food variety may not only impact upon body mass as a factor of increased consumption but may also impact upon sensory-specific satiety. Thus, a diet high in variety may influence the devaluation of a particular food associated with a CS, and also constrain behavioral control based on incentive value.

Effects of food variety on sensory specific satiety have been little explored, particularly in animal models. In this study we sought to establish the impact of a rodent model of diet-induced obesity that utilizes a diet reflective of a modern obesogenic diet (Hansen et al., 2004; Martire et al., 2013) upon CS-outcome associations and the expression of specific satiety.

## MATERIALS AND METHODS

### EXPERIMENT 1A – IMPACT OF OUTCOME-DEVALUATION ON PAVLOVIAN CONDITIONED APPROACH

#### Subjects

Subjects were 32 experimentally naïve male Sprague–Dawley rats obtained from Animal Resources Center (Perth, WA, Australia). Rats were 6 weeks old at arrival and weighed 230–270 g. They were housed in groups of four in plastic cages (36 cm wide × 26 cm high × 62 cm deep) located in a temperature and humidity controlled room (Mean temperature 20 ± 2°C, humidity 50 ± 5%) on a 12 h light: 12 h dark cycle (lights on at 07:00). Testing was carried out during the light phase of the cycle, between 08:00 and 13:00. During testing, rats were water restricted (2 h access per day between 13:00 and 15:00). Food was available ad lib throughout testing; in the control diet condition this was standard laboratory chow and in the cafeteria diet condition this was laboratory chow supplemented with a range of foods eaten by people (see below). During behavioral training water access was restricted within the home cages to 3 h per day following training sessions. All experimental procedures were approved by the Animal Care and Ethics Committee at the University of New South Wales and were in accordance with the National Institutes of Health Guidelines for the Care and Use of Laboratory Animals (revised 1996).

#### Diet

Rats were handled daily and allowed to acclimatize to housing for one week. Standard lab chow and water was available ad lib. Following this acclimatization, rats were randomly allocated to either standard lab chow (Group Chow) or a high fat cafeteria diet (Group Cafeteria) condition (*N* = 16 per group). Standard chow provided 11 kJ/g, 12% energy as fat, 23% protein, and 65% as carbohydrate (Gordon’s Specialty Stockfeeds, NSW, Australia). The cafeteria diet consisted of lab chow supplemented with four commercially available foods. Rats were given a standardized selection of foods each day which previous studies from our laboratory show are equally well preferred; each day foods consisted of two savory items (e.g., pies, dim sims) and two sweet items (e.g., cookies, cakes, biscuits). This diet provided an average of 13.8 kJ/g, 33% energy as fat, 11% protein, and 56% as carbohydrate, in addition to that provided by the standard laboratory chow. Rats consuming this cafeteria diet obtain approximately four times the energy and have a fat mass 2.5times greater than control rats fed standard laboratory chow (Martire et al., 2013). The cafeteria diet was presented inside the home cages daily, at 13:00 h; the cafeteria foods were available *ad libitum* and changed daily to allow measurements of energy intake and prevent spoilage. Water was available *ad libitum*. Energy intake and body weight were measured once per week. On the intake measurement days foods were consistent across weeks, rats received beef pie (8.55 kJ/g, Coles, Australia), Dim Sims (7.9 kJ/g, Coles, Australia), jam roll (14.9 kJ/g, Coles, Australia), lamington cakes (13.8 kJ/g, Coles, Australia) in addition to standard lab chow (11 kJ/g).The amount consumed was the difference between the weight of the food allocated to a cage and that remaining 24 h later. Energy intake for each cage was calculated using the known energy content (kJ/g) and macronutrient content (% protein, carbohydrate, and fat) of each food. This was divided between the numbers of rats in the cage (*N* = 4) to obtain mean energy consumption per rat. Rats were exposed to the cafeteria diet for 2 weeks prior to Pavlovian conditioned approach training.

#### Apparatus

Rats received Pavlovian training in four chambers (30 cm wide, 21 cm high, and 24 cm deep) located in sound-attenuating boxes (Med Associates, St Albans, VT, arranged in a two-by-two array in a room which remained dark throughout the experiment. Each chamber consisted of three walls and a ceiling, with the door serving as the fourth wall. The ceiling, door and back wall were made from clear Perspex and the left and right walls were made from stainless steel. The floor of each chamber consisted in stainless steel rods (4.8 mm in diameter, spaced 16 mm apart). Each chamber was illuminated by a 3W house light located at the top center of one wall and a speaker was fitted into this wall. The opposite walls of the chambers were fitted with a recessed magazine with two metal spouts to allow separate delivery of solutions via pumps. The solutions used were 10% (w/v) sucrose flavored with 0.05% (w/v) cherry Kool Aid, and 10% (w/v) maltodextrin flavored with 0.05% (w/v) grape Kool Aid.

An infra-red camera located in the sound attenuating box allowed behavior to be recorded to DVD for subsequent scoring of magazine entry behavior. A computer equipped with MED-PC software (version IV; Med Associates Inc.) controlled the stimulus and outcome presentations. The stimuli consisted of a 2 kHz 78 dB pure tone and a 75 dB white noise measured by a sound level meter (Dick Smith Electronics, Australia).

#### Procedure

**FIGURE 1:**
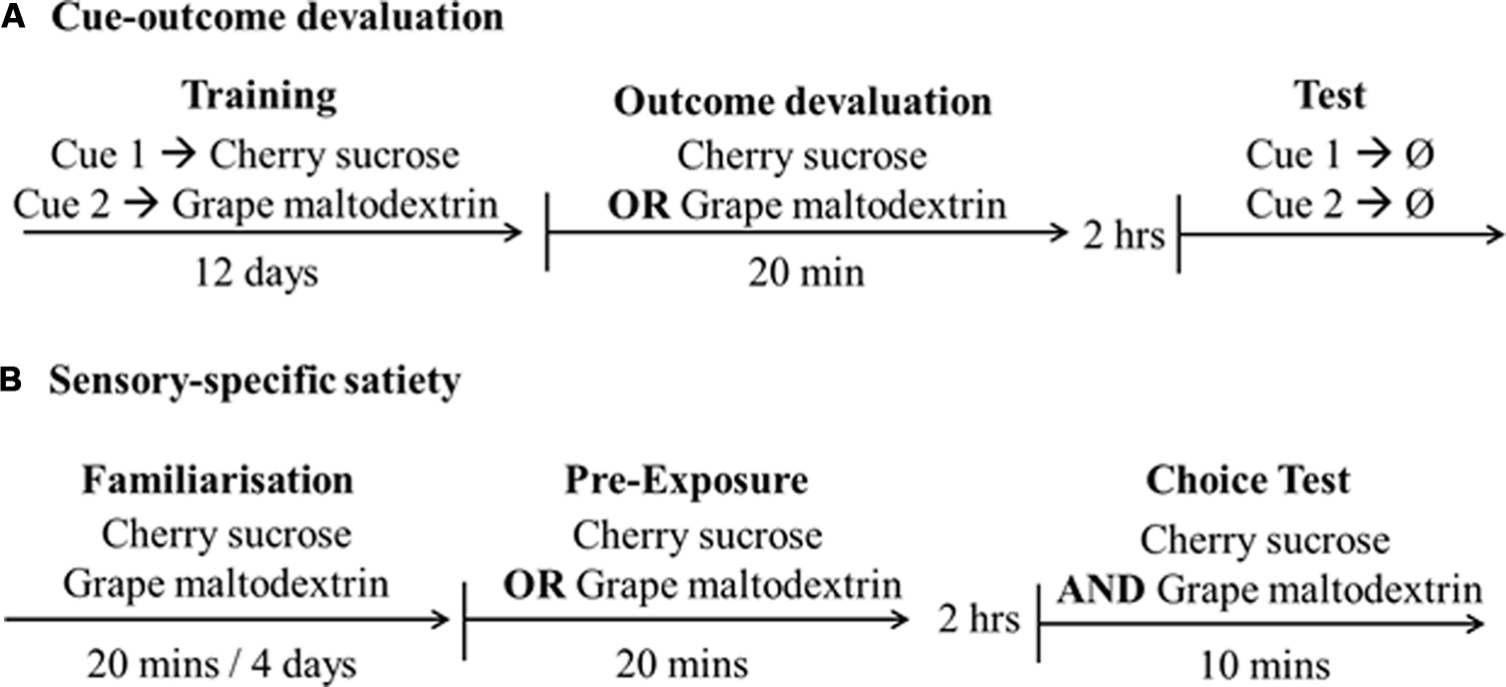
**Design and timeline of the studies. (A)** Cue-outcome devaluation and **(B)** Sensory-specific satiety, indicating outcomes [cherry sucrose, grape maltodextrin, or no reward (Ø)].

***Pavlovian conditioning.*** Rats were trained to consume the solutions from the magazine during a 30 min session, repeated over 2 days. Pavlovian training was carried out over 12 days (one session per day) during which two discriminable auditory stimuli (CS): white noise or tone – presented 10 times each in a randomized order each session for 15 s. Each CS (noise or tone; counterbalanced across rats) was consistently followed by presentation of one of the solutions, e.g., tone followed by 0.1 ml of cherry flavored sucrose [outcome 1 (O1)] and noise followed by 0.1 ml of grape flavored maltodextrin[outcome 2 (O2)] as shown in **Figure 1A**. Each stimulus presentation was separated by a variable inter-trial interval (ITI; mean 90 s) and a PreCS (15 s).

***Outcome devaluation.*** Devaluation consisted in allowing the rats to drink to satiety one of the solutions (O1 or O2). Rats were placed in individual plastic cages (30 cm wide, 25 cm high, 45 cm deep) with a wire mesh ceiling and a sawdust covered floor. Rats were presented with either 50 ml of grape maltodextrin or cherry sucrose solution in a measuring tube bottle with a ball bearing drinking spout. One half of the rats were devalued with outcome O1, the other half with O2. Therefore, each rat was devalued with an outcome associated and not associated with each auditory cue. Rats were returned to their home cages for 2 h and were then tested.

***Test.*** Magazine activity was measured by head entry into the recessed magazine during non-reinforced auditory CS presentations. There were three randomized presentations of the white noise and of the tone, each presentation being 15 s in duration and each presentation separated by a variable stimulus free period ITI (mean = 90 s) and 15 s PreCS. Two observers, “blind” with respect to the assignment of groups, scored the amount of time each rat spent entering the magazine during each CS presentation. The correlation between their scores was high, *r* = 0.82.

### EXPERIMENT 1B – SENSORY-SPECIFICSATIETY IN CAFETERIA DIET EXPOSED RATS

#### Subjects and apparatus

Rats from Experiment 1A were tested for consumption in individual plastic cages (30 cm wide, 25 cm high, 45 cm deep) with a wire mesh ceiling and a sawdust covered floor 1 week after finishing Experiment 1A. Two palatable solutions were used as described in Experiment 1A; 10% (w/v) sucrose flavored with 0.05% (w/v) cherry Kool Aid and 10% (w/v) maltodextrin flavoured with 0.05% (w/v) grape Kool Aid dissolved in tap water. These solutions were matched for energy content (1680 kJ per 100 ml)and previously demonstrated to be equally preferred and discriminable (Reichelt et al., 2013). Rats were presented with 50 ml of the solutions in a plastic measuring tube bottle with a ball bearing drinking spout.

#### Procedure

As shown in **Figure 1B** rats were familiarized with the solutions in the individual testing chambers over a 2 day period. Rats received a ball spouted bottle containing 50 ml of each solution separately in a 20 min session across the 2 days. Rats received two tests on consecutive days. Rats were placed in the testing chambers and allowed to freely consume one solution for 20 min. This solution was the cherry flavored sucrose for half of the rats and grape flavored maltodextrin for the remainder. They were then returned to their home cage for 2 h. The rats were then returned to the individual testing chambers for 10 min and presented with two bottles; one containing the solution which the rats had drank 2 h previously and the second bottle containing the other solution. Volumes consumed were recorded as ml. On Day 1 rats were exposed to a solution (e.g., cherry sucrose) and then tested with both solutions presented simultaneously (cherry sucrose and grape maltodextrin). On Day 2, rats were exposed to the alternative solution (grape maltodextrin) and then tested with both solutions simultaneously. Thus, a within-subject comparison could be made in a fully counterbalanced manner.

### EXPERIMENT 2 – EXPRESSION OF SENSORY-SPECIFIC SATIETY FOLLOWING LIMITED PRE-EXPOSURE VOLUME

#### Subjects

Subjects were 24 naïve adult male Sprague-Dawley rats obtained from Animal Resources Centre (Perth, Western Australia). They weighed between 435–510 *g* and were housed in the manner described previously with *ad libitum* access to water and standard chow.

#### Apparatus

Individual consumption cages were identical to that described in Experiment 1.The two solutions used in this experiment were 10% (w/v) sucrose and 14% (w/v) vanilla Sustagen (Nestle) dissolved in tap water. These solutions were used in Experiments 2 and 3 to assess the reliability of effects observed with cherry flavored sucrose and grape flavored maltodextrin solution. Solutions were matched for energy content of 1680 kJ per 100 ml; pilot studies indicated the solutions were equally preferred and discriminable.

#### Procedure

The rats were familiarized with these solutions in a 2 day pilot study, where the rats were exposed to one solution (e.g., sucrose) on day one, and the other solution (e.g., vanilla Sustagen) on day two. One week later they received one test of sensory-specific satiety. The rats were allowed to consume a limited volume of an outcome during pre-exposure in order to assess whether the smaller volume consumed by cafeteria diet fed rats was capable of inducing sensory-specific satiety. The rats were presented with 10 ml of either solution during pre-exposure for 20 min. Rats were returned to their home cages for 120 min. At test, rats were presented with a two bottle choice test as described previously.

### EXPERIMENT 3 – SENSORY-SPECIFIC SATIETY IN CAFETERIA DIET WITHDRAWN RATS

#### Subjects and diet

Adult male Sprague–Dawley rats (*N* = 24), obtained from Animal Resources Center (Perth, Western Australia), were used as subjects and housed as described above. Half of the rats (*N* = 12) were maintained on the cafeteria diet described previously for 10 weeks, and the remainder were fed standard chow. After 10 weeks the cafeteria diet was withdrawn from the rats and replaced with standard chow for 1 week prior to testing.

#### Apparatus

The two solutions used in this experiment were 10% (w/v) sucrose and 14% (w/v) vanilla Sustagen (Nestle) dissolved in tap water (as Experiment 2). Rats were presented with 50 ml of the solutions in a plastic measuring tube bottle with a ball bearing drinking spout. Rats were tested for consumption in the individual plastic and wire cages described previously.

#### Procedure

The rats were already familiar with these solutions from a pilot study that tested whether consumption of the two solutions was comparable across diet groups across a 2 day period where the rats were exposed to one solution (e.g., sucrose) on day one, and the other solution (e.g., vanilla Sustagen) on day two, so both groups were matched in their history of consuming each of the test solutions. Rats were tested a week later for specific satiety over a 2 day period as described in Experiment 1B.

#### Statistical analysis

Results are expressed as mean ± SEM. Data were analyzed using IBM SPSS Statistics 22 and GraphPad Prism 6. Data were analyzed using repeated measures analysis of variance (ANOVA), analysis of covariance (ANCOVA), or independent *t*-test where appropriate. *Post hoc* tests were performed where significant interactions were observed, and controlled by Bonferroni correction. The critical *F* was chosen to maintain the type 1 error rate at less than 0.05.

## RESULTS

### EXPERIMENT 1A – IMPACT OF OUTCOME-DEVALUATION ON THE CONTROL OF PAVLOVIAN RESPONDING

#### Body weight

**FIGURE 2:**
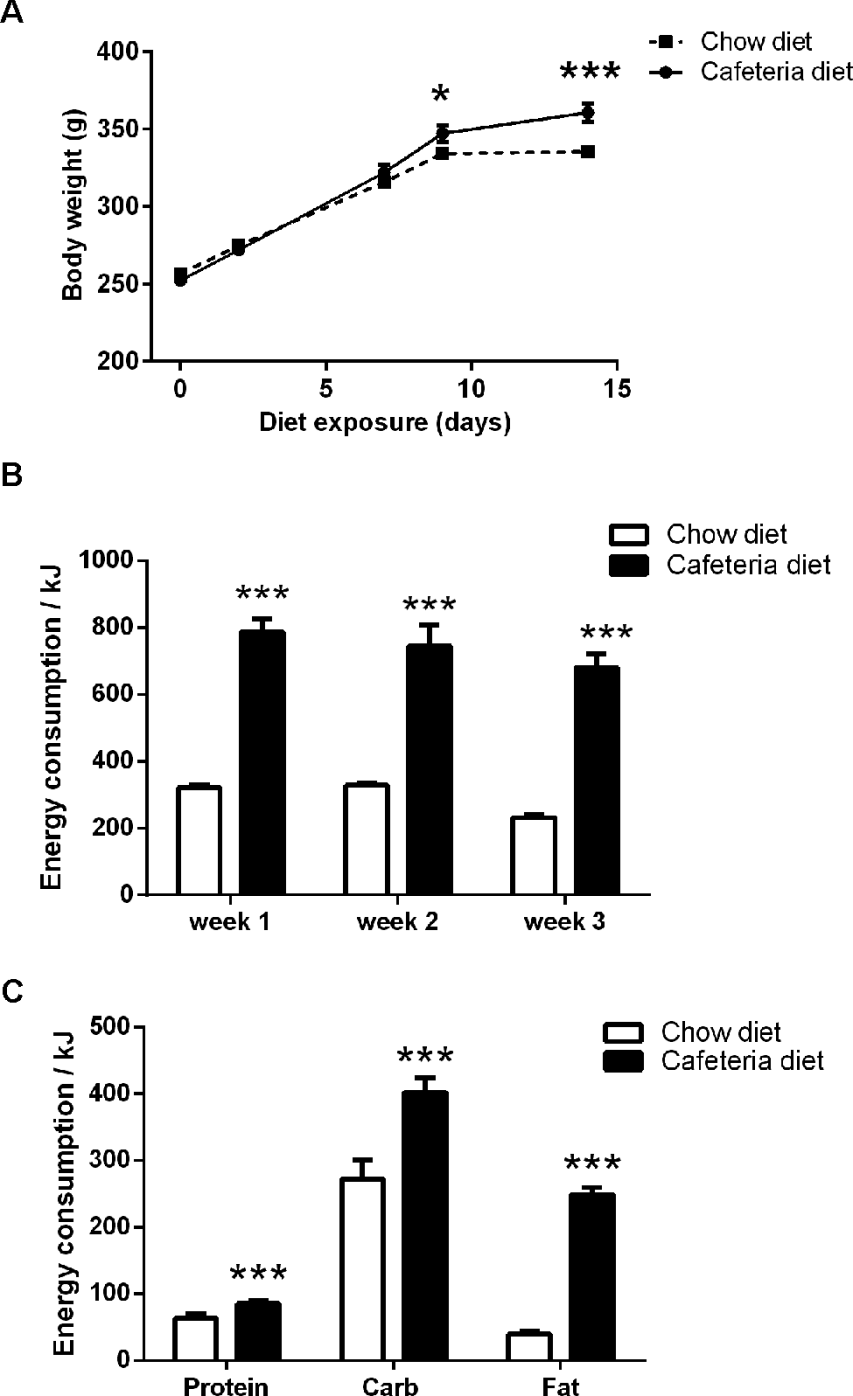
**(A)** Body weight of cafeteria (*N* = 16) and chow (*N* = 16) diet rats. **(B)** Total energy intake over 24 h (kJ/rat). **(C)** Macronutrient intake over 24 h (protein, carbohydrate, and fat) as energy (kJ/rat). Data presented as mean (±SEM). *p < 0.05, ***p ≤ 0.001 compared to chow, Bonferroni corrected.

Rats exposed to the cafeteria diet for 14 days had significantly greater body weights than chow fed animals (**Figure 2A**). This was confirmed by repeated measures ANOVA with between subject factors of diet (cafeteria, chow) and within subject factor of diet exposure (days). This revealed a significant main effect of diet exposure, *F*(4,120) = 1003.9, *p* < 0.001, no main effect of diet, *F*(1,30) = 2.0, *p* = 0.165, and a significant interaction between diet exposure × diet, *F*(4,120) = 21.9, *p* < 0.001. Inspection of the simple main effects indicated that all rats increased in weight across exposure to cafeteria and chow diets, (*F*’s > 141.1, *p* < 0.001). However, cafeteria diet fed rats were significantly greater in body mass after 14 days exposure, *F*(1,30) = 13.2, *p* = 0.001.

#### Energy consumption

Rats fed the cafeteria diet consumed, on average, 2.5 times more energy (as kJ) than the chow fed rats, as shown in **Figure 2B**. Repeated measures ANOVA between subject factors of diet (cafeteria, chow) and within subject factor of diet exposure (week) revealed a significant main effect of diet, *F*(1,3) = 433.4, *p* < 0.001, no significant main effect of diet exposure, *F*(2,6) = 3.5, *p* = 0.097, and no significant diet x exposure interaction, *F* < 1. As shown in **Figure 2C**, rats fed the cafeteria diet consumed significantly more energy (kJ) as protein, (*t* = 8.4, *df* = 6, *p* < 0.001), carbohydrate, (*t* = 8.0, *df* = 6, *p* < 0.001), and fat, (*t* = 21.7, *df* = 6, *p* < 0.001), than chow fed rats.

#### Training

**FIGURE 3:**
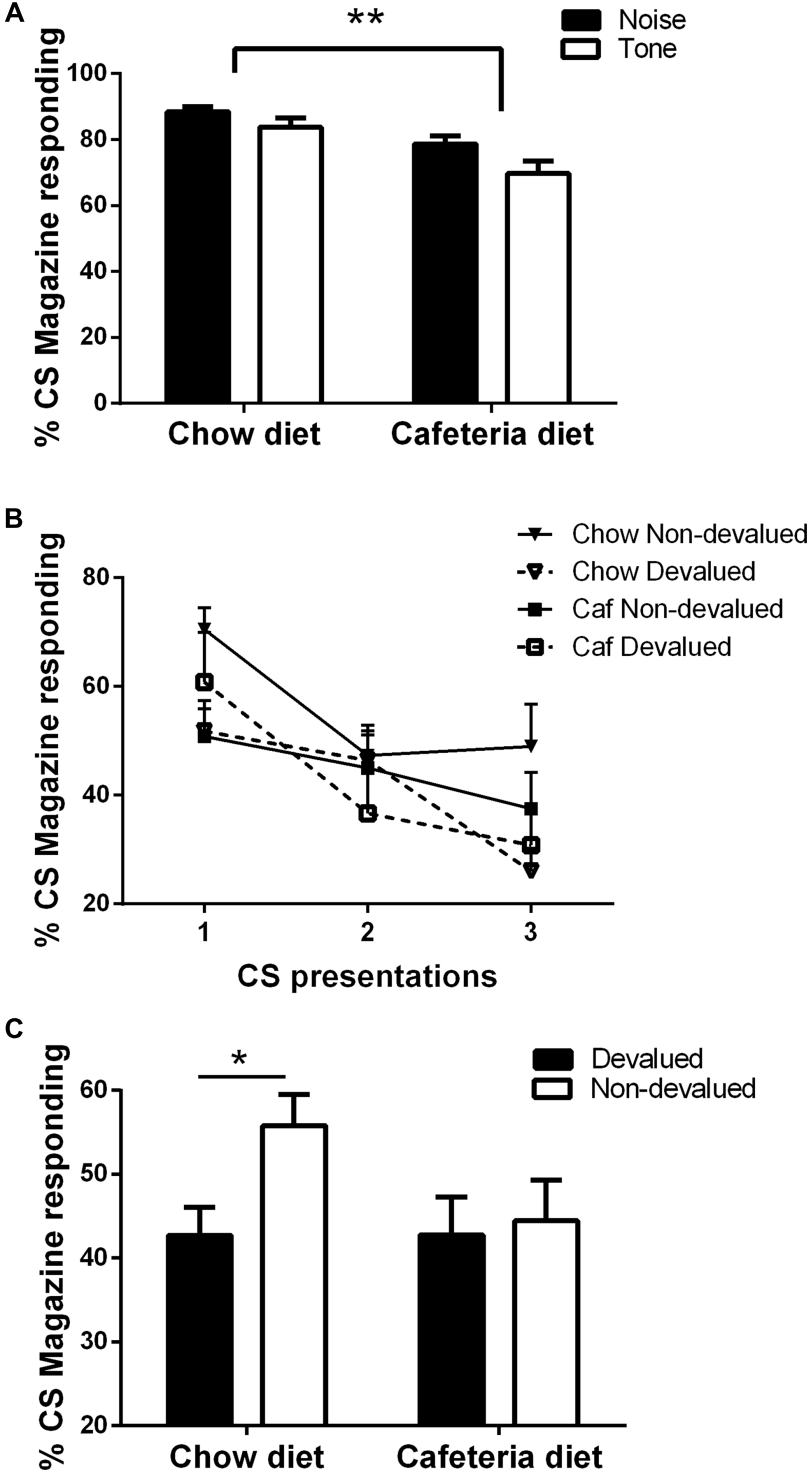
**(A)** Magazine responding in the final training session; **(B)** Magazine responding (Mean CS1-3) at test and **(C)** Mean magazine responding at test across all CSfor chow diet rats (*N* = 14) and cafeteria diet rats (*N* = 15). Data presented as mean (±SEM). *p < 0.05, **p < 0.01 Bonferroni corrected.

As illustrated in **Figure 3A**, both cafeteria diet and chow fed rats learned about the CS–US relations, as shown by % time spent making magazine responses during the 15 s CS presentations on the last day of training relative to the PreCS. This was confirmed by ANOVA with within-subject factors of CS (noise, tone), and between-subject factors of diet (cafeteria, chow), which revealed a significant main effect of CS [*F*(1,27) = 8.5, *p* < 0.01] and diet [*F*(1,27) = 13.4, *p* < 0.01], indicating that the chow rats spent a greater % of time in the magazine during the CS presentations, and that these rats responded more to the noise than tone. There were no statistically significant two-way interactions between CS × diet (*F* < 1). Chow and cafeteria fed rats responded equally during the PreCS periods (Mean % PreCS magazine responses: chow = 8.1 (±2.2), cafeteria = 10 (±3.6), independent samples *t*-test *t* < 1. Furthermore, there was no difference between responding to the CS based on its associated outcome pairing, confirmed by ANOVA demonstrating no significant main effect of counterbalancing [*F*(1,25) = 1.8, *p* = 0.197]. No interactions were significant (*F*’s < 4.03).

#### Outcome devaluation

Three rats were excluded from the statistical analysis (two from the chow and one from the cafeteria diet condition) due to not consuming the solution during the outcome devaluation or failing to make magazine responses during the extinction test. Chow fed rats consumed a significantly greater volume of the devalued outcome during pre-exposure [Mean (±SEM): Cafeteria = 8.93 ml (0.79 ml), Chow = 14.1 ml (0.85 ml); independent samples *t*-test *t* = 4.44, *df* = 27, *p* < 0.001].

#### Test

The test session was divided into three time points, each consisting of a presentation of the CS associated with the devalued outcome and the CS associated with the non-devalued outcome. As shown in **Figure 3B**, chow fed rats generally responded more to the CS associated with the non-devalued outcome, whereas cafeteria fed rats responded more to the CS associated with the devalued outcome during the first 2 CS presentations (time point 1which includes CS associated with devalued and non-devalued outcome). Analysis of % magazine responding across the three time points (CS associated with devalued and non-devalued outcome) by repeated measures ANCOVA with within subjects factors of devaluation (devalued, non-devalued) and time point (1–3), between subject factor of diet (cafeteria diet, chow), and covariate of volume consumed during outcome devaluation (consumption) revealed significant main effect of time point [*F*(2,44) = 4.287, *p* < 0.001] and devaluation [*F*(1,22) = 6.3, *p* < 0.05], but no significant main effect of diet [*F*(1,22) = 2.73, *p* = 0.113] or consumption [*F*(1,22) = 1.16, *p* = 0.29]. Significant interactions were observed between devaluation × diet [*F*(1,22) = 8.66, *p* < 0.01], time × devaluation [*F*(1,22) = 3.97, *p* < 0.05], time × devaluation × consumption [*F*(2,44) = 3.86, *p* < 0.05] and time × devaluation × diet [*F*(2,44) = 3.29, *p* < 0.05], no other interactions were significant (*Max F* = 3.37). Simple main effects were used to break down the devaluation × diet interaction. As shown in **Figure 3C**, no significant effect of devaluation was observed in cafeteria diet fed rats (*F* < 1), however, a significant effect of devaluation was observed in chow diet fed rats [*F*(1,26) = 8.662, *p* < 0.01].

### EXPERIMENT 1B – SENSORY-SPECIFIC SATIETY IN CAFETERIA DIET EXPOSED RATS

#### Body weight

Rats assigned to the cafeteria and chow diets continued to be exposed to the allocated diet throughout training and testing. At test, rats in the cafeteria diet group were significantly heavier than chow fed rats [Mean (±SEM): Cafeteria = 530 g (13.5 g), chow = 457.9 g (7.8 g), *t* = 4.6, *df* = 30, *p* < 0.001].

### SENSORY-SPECIFIC SATIETY TEST

#### Familiarization

**FIGURE 4:**
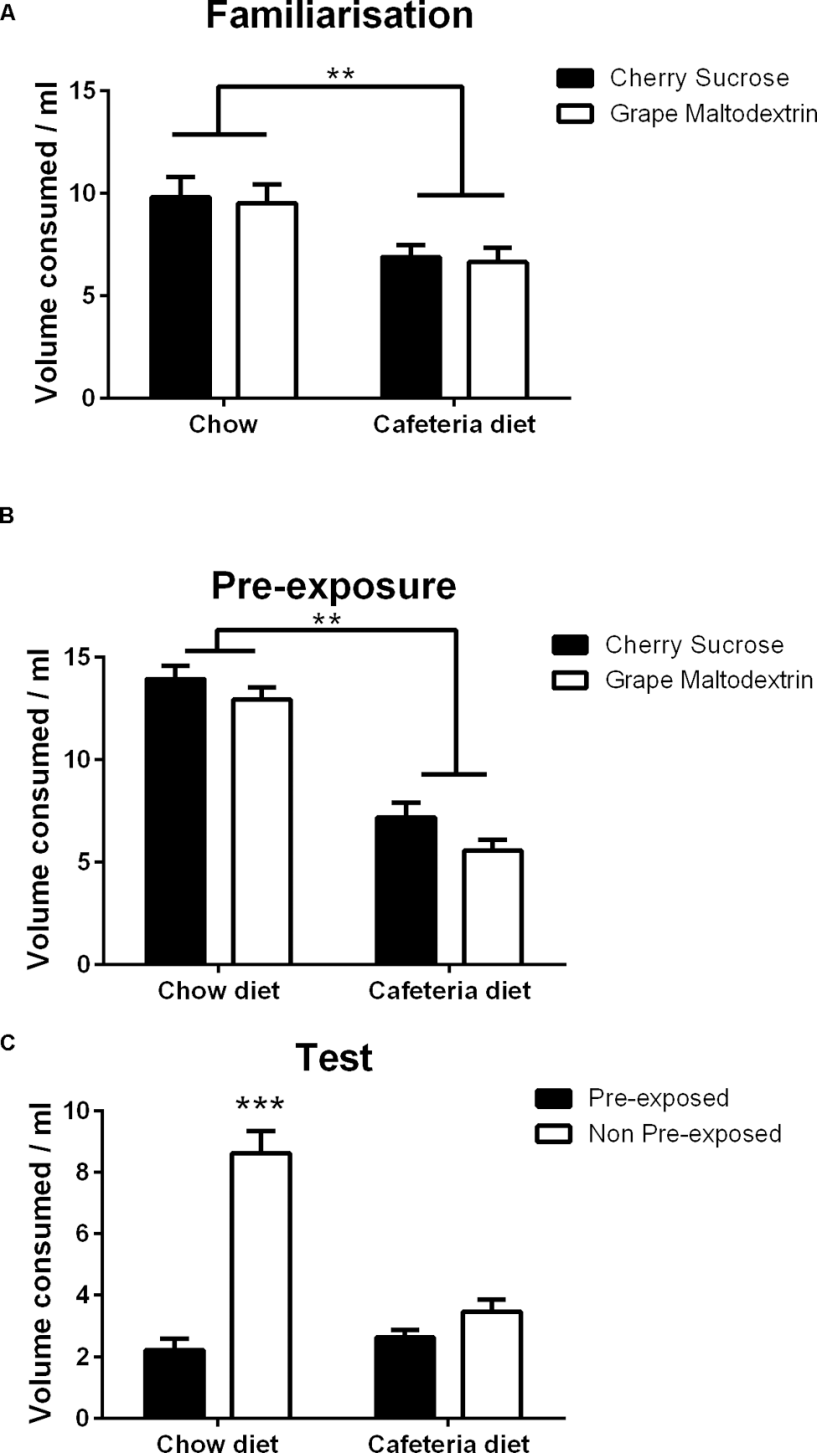
**Consumption of sample solutions during (A) Familiarization to the two solutions, (B) Pre-exposure to the solutions prior to test, (C) Sensory-specific satiety test indicating the mean volume consumed of the pre-exposed and non-pre-exposed solutions during two bottle choice testing by chow (*N* = 16) and cafeteria (*N* = 16) diet fed rats.** Data presented as mean (±SEM). **p < 0.01, ***p < 0.001. Bonferroni corrected.

As shown in **Figure 4A**, chow fed rats consumed a greater volume than cafeteria diet fed rats, but both groups drank similar amounts of both solutions. These observations were confirmed by a repeated measures ANOVA with within subject factors of solution (cherry sucrose, grape maltodextrin) and between subject factor of diet (cafeteria, chow), which revealed a significant main effect of diet [*F*(1,30) = 13.6, *p* < 0.001, but no significant main effect of solution (*F* < 1) or solution × diet interaction (*F* < 1).

#### Pre-exposure

Rats consumed similar volumes of each solution, and chow fed rats consumed a greater volume than cafeteria fed rats as shown in **Figure 4B**. This observation was confirmed by ANOVA with within subject factors of solution (cherry sucrose, grape maltodextrin) and between subject factor of diet (cafeteria, chow), which revealed a significant main effect of solution [*F*(1,30) = 6.2, *p* < 0.05], which was due to greater intake of the cherry sucrose than the grape maltodextrin, a significant main effect of diet [*F*(1,30) = 102.6, *p* < 0.001], and no significant solution diet × interaction (*F* < 1).

#### Two bottle choice test

Chow fed rats consumed a greater volume of the non-pre-exposed solution, indicating sensory-specific satiety, whereas cafeteria diet rats consumed similar volumes of both the pre-exposed and non-pre-exposed solution, indicating the absence of sensory-specific satiety, as shown in **Figure 4C**. This observation was confirmed by a repeated measures ANCOVA with within subject factors of exposure (pre-exposed, non-pre-exposed), between subject factor of diet (cafeteria, chow) and covariate of volume consumed during pre-exposure, which revealed a significant main effect of exposure [*F*(1,29) = 4.598, *p* < 0.05], no significant main effect of diet [*F*(1,29) = 3.233, *p* = 0.083], no significant effect of pre-exposure volume [*F*(1,29) = 1.468, *p* = 0.235]. A significant exposure × diet interaction was observed [*F*(1,29) = 11.777, *p* < 0.01], but no significant interaction between exposure and volume consumed during pre-exposure (*F* < 1). Simple main effects analysis of the solution exposure × diet interaction indicated that there was no effect of exposure in the cafeteria diet fed rats (*F* < 1), but a significant effect of exposure in chow fed rats [*F*(1,29) = 40.107, *p* < 0.001]. Thus, cafeteria diet fed rats treated the pre-exposed and non-pre-exposed solutions as equivalent, indicative of impaired sensory-specific satiety.

Preference between the two solutions consumed at test was equivalent, indicated by similar volumes consumed [Chow diet – Means (±SEM): cherry sucrose = 11.4 ml (0.78 ml), grape maltodextrin = 10.3 ml (0.89 ml). Cafeteria diet – Means (±SEM): cherry sucrose = 6.6 ml (0.97 ml), grape maltodextrin = 5.6 ml (0.58 ml)]. This observation was confirmed by repeated measures ANOVA with within subject factor of solution (cherry sucrose, grape maltodextrin) and between subject factor of diet (cafeteria, chow), with no significant main effect of solution [*F*(1,30) = 1.569, *p* = 0.22], a significant main effect of diet [*F*(1,30) = 31.2, *p* < 0.001], and no significant solution × diet interaction (*F* < 1).

### EXPERIMENT 2 – EXPRESSION OF SENSORY-SPECIFIC SATIETY FOLLOWING LIMITED PRE-EXPOSURE VOLUME

#### Pre-exposure

Rats consumed equal volumes of each solution [Mean (±SEM) = sucrose 9.41 ml (0.36 ml), vanilla 9.16 ml (0.37 ml), independent samples *t*-test: *t* < 1].

#### Two bottle choice test

Chow fed rats consumed a greater volume of the non-pre-exposed solution, indicative of intact sensory-specific satiety [Means (±SEM): pre-exposed solution = 3.87 ml (0.69 ml), non-pre-exposed solution = 10ml (0.78 ml), paired samples *t*-test: *t* = 4.95, *df* = 23, *p* < 0.001]. Thus, rats pre-exposed to a limited volume of up to 10 ml demonstrated intact sensory-specific satiety. It can therefore be suggested that a smaller volume of solution during pre-exposure was sufficient to produce sensory-specific satiety at test in chow fed rats.

### EXPERIMENT 3 – SENSORY-SPECIFIC SATIETY IN CAFETERIA DIET WITHDRAWN RATS

#### Body weight

At test, rats withdrawn from the cafeteria diet were still significantly heavier than rats only fed chow [Mean (±SEM): Ex-Cafeteria = 696.7 g (11 g), chow = 582.3 g (10.9 g), *t* = 7.419, *df* = 22, *p* < 0.001].

#### Pre-exposure

Rats consumed similar volumes of each solution, and chow fed rats consumed a greater volume than rats previously cafeteria diet fed (Mean (±SEM) Ex-Cafeteria = sucrose 16 ml (0.83 ml), vanilla 16.08 ml (1.4 ml), Chow = sucrose 21.08 ml (1.05 ml), vanilla 18.42 ml (1.07 ml). This observation was confirmed by ANOVA with within subjects factors of solution (sucrose, vanilla) and between subjects factor of diet (ex-cafeteria, chow), which revealed no significant main effect of solution [*F*(1,22) = 1.4, *p* = 0.257], a significant main effect of diet [*F*(1,22) = 11.1, *p* < 0.01], and no significant solution × diet interaction [*F*(1, 22) = 1.0, *p* = 0.497].

#### Two bottle choice test

**FIGURE 5:**
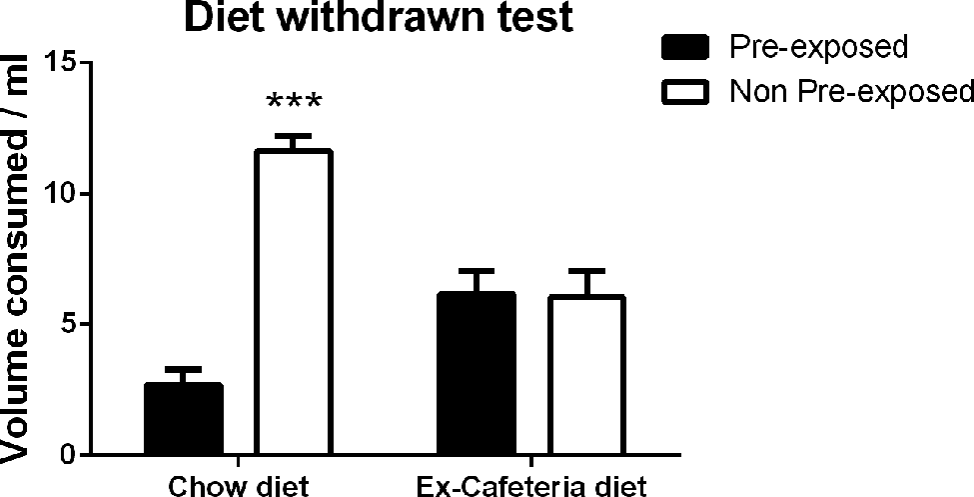
**Two bottle choice test of sensory-specific satiety following pre-exposure to palatable solutions in rats 1 week after withdrawal of the cafeteria diet (*N* = 12) and chow fed control rats (*N* = 12).** Data presented as mean (±SEM). ***p < 0.001.Bonferroni corrected.

Rats only ever fed chow consumed a greater volume of the non-pre-exposed solution, indicating sensory specific satiety, whereas rats withdrawn from the cafeteria diet and fed chow consumed similar volumes of both the pre-exposed and non-pre-exposed solutions, indicating the absence of sensory specific satiety, as shown in **Figure 5**. This observation was confirmed by ANCOVA with within subject factors of exposure (pre-exposed, non-pre-exposed), between subject factor of diet (ex-cafeteria, chow) and a covariate of pre-exposure volume consumed (pre-exposure) which revealed no significant main effect of exposure (*F* < 1), a significant main effect of diet [*F*(1,21) = 3.56, *p* < 0.05], and a significant exposure × diet interaction [*F*(1,21) = 13.97, *p* = 0.001]. There was no main effect of pre-exposure volume as a covariate [F(1,21) = 3.56, *p* = 0.073], or exposure x pre-exposure interaction (*F* < 1). The simple main effects analysis indicated that there was no effect of exposure in the cafeteria diet fed rats (*F* < 1), however, there was a significant effect of exposure in chow fed rats [*F*(1,21) = 32.564, *p* < 0.001]. Thus, rats previously consuming a cafeteria diet still demonstrated impaired sensory-specific satiety 1 week following withdrawal of the cafeteria diet, indicative of a prolonged effect of the cafeteria diet.

In addition, there was no preference between the two different solutions consumed at test. ANOVA, with within subject factors of solution (sucrose, vanilla) and between subject factor of diet (ex-cafeteria, chow), confirmed that there was no significant main effect of solution [*F*(1,22) = 1.6, *p* = 0.22], diet [*F*(1,22) = 3.6, *p* = 0.072], and no significant solution × diet interaction (*F* < 1).

## DISCUSSION

The results of the present experiments show that rats fed a cafeteria diet, containing foods eaten by people, were impaired in both the value-driven guidance of food seeking responses by cues associated with palatable solutions and in the expression of sensory-specific satiety. Moreover, this impairment in the expression of sensory specific satiety among rats fed the cafeteria diet was also present when this diet was removed and replaced with standard chow for 1 week. Finally, this impairment did not appear to be due to differences between the amounts consumed of the pre-exposed solution as chow fed rats exhibited sensory specific satiety independently of the amounts consumed of the pre-exposed solution, as shown by our analysis of covariance.

Neuroimaging studies in humans and non-human primates link the OFC to hedonic processing and the alignment of eating with the value of a food (Kringelbach et al., 2003; Kringelbach, 2005). Furthermore, primate studies indicated that consuming a food to satiety decreased neural responsiveness in the OFC, and this responsiveness is recovered upon the presentation of a new food (Rolls et al., 1990). Thus, the OFC has been implicated as a key neural region in the evaluation of the pleasurable aspects of palatable foods and in encoding the sensory attributes of these values. In light of the observation that sensory-specific satiety is impaired in rats fed a cafeteria diet, and evidence that the OFC is a critical region involved in integrating an updating value-based information about reward-predictive cues (Delamater, 2007; Ostlund and Balleine, 2007; Clark et al., 2012), we suggest that that the outcome-value encoding systems are disrupted following exposure to palatable foods in cafeteria diets. An implication of this suggestion is that presentation of a novel food to cafeteria-fed rats would fail to recover neural responses in the OFC and that this may disrupt the selection of a different food in the case of sensory specific-satiety and the updating of the incentive value of a food outcome to direct conditioned responding.

Rats fed a cafeteria diet responded to two cues predictive of separate palatable reward during training. However, following devaluation of one of these outcomes by specific satiety, cafeteria fed rats did not modulate magazine responding in accordance with the incentive value of the outcome. Our results indicate that chow rats were sensitive to devaluation, but cafeteria diet rats were not when analysis was carried out across all trials. However, it is worth noting that the magnitude of the devaluation effect changed across trials. This indicates that consumption of an obesogenic cafeteria diet may impact upon brain regions involved in the formation of stimulus-outcome associations and incentive value, such as the basolateral amygdala (BLA), striatum and OFC, as described previously. Johnson et al. (2009) reported that the BLA plays a critical role in devaluation performance after multiple-reinforcer Pavlovian conditioning. However, Johnson et al. (2009) utilized taste aversion as opposed to specific satiety to modulate the value of the appetitive outcomes, and also demonstrated that post-training BLA lesions disrupted the expression of incentive value-controlled behaviors. Similarly, Balleine et al. (2011) and Ostlund and Balleine (2007) found that OFC lesions disrupted the influences of Pavlovian stimuli during outcome-specific Pavlovian-instrumental transfer. The influence of outcome-related stimuli on choice involves a larger circuit including the OFC, the striatum, and the amygdala. In particular, the central nucleus of the amygdala has been shown to be necessary for conditioned approach to cues measured by sign-tracking behaviors (Gallagher et al., 1990; Parkinson et al., 2000); in addition, sensory-specific satiety is disrupted by transient inactivation of the central nucleus of the amygdala (Ahn and Phillips, 2002). Therefore, our observation of impaired sensory-specific satiety and cue-outcome associations may indicate that the cafeteria diet also affected central amygdala function.

The failure to detect an effect of the devalued outcome on the magazine approach responses elicited by its CS associate is consistent with human neuroimaging studies demonstrating differential activation of reward neurocircuitry (particularly the mesocorticolimbic dopamine system) by food-associated cues in obese subjects (Stoeckel et al., 2008, 2009; Jastreboff et al., 2013). Previous devaluation studies in rats have demonstrated that the BLA has a fundamental role in the maintenance of detailed sensory-specific outcome representations, allowing the integration of new information about outcome value into existing associative structures (Ostlund and Balleine, 2007). Furthermore, regions of the striatum, in particular the ventrolateral (Lelos et al., 2011), dorsomedial, and dorsolateral striatum (Corbit and Janak, 2010), have been implicated in Pavlovian outcome devaluation, as has the NAc core and shell (Corbit et al., 2001). However, OFC and BLA lesions have no detectable effects on the formation or flexible use of sensory-specific flavor-nutrient associations in a devaluation task (Scarlet et al., 2012), or consumption tests following devaluation (Corbit et al., 2001; Corbit and Janak, 2010; Lelos et al., 2011). Similarly, the NAc core and shell has been shown to be necessary for the control of Pavlovian conditioned responding following devaluation by LiCl induced nausea (Singh et al., 2010). These data suggest that NA core and shell are part of a circuit necessary for the use of cue-evoked information about expected outcomes to guide behavior, particularly involving regions such as the OFC and BLA that project to the NAc.

This is the first study to demonstrate impairment in the expression of sensory-specific satiety in rats fed a cafeteria diet, which may underpin maladaptive eating behaviors in obesity. Studies investigating whether obesity affects sensory-specific satiety in people have reported mixed results. Tey et al. (2012) found that people with a greater body mass index and fat mass showed decreased sensory-specific satiety at baseline. This study also showed that people who regularly consumed the same three energy dense snack foods showed a reduction in sensory-specific satiety over time, so eating of these snack foods became less influenced by the previously consumed foods. In contrast, limiting the variety of snack foods available resulted in decreased hedonic ratings of snack foods and reduced intake in both normal weight and overweight adult participants, indicative of long term sensory-specific satiety (Raynor et al., 2006). In contrast, a previous study with obese and normal weight participants showed no differences in sensitivity to sensory-specific satiety (Snoek et al., 2004).

In this study, we observed that cafeteria diet rats consumed equal volumes of the pre-exposed and non-pre-exposed solutions. This is an intriguing observation, as the failure of cafeteria diet fed rats to consume more of the novel solution may be construed as being protective against overeating and thus long term weight gain. Consumption of a varied diet of palatable foods that appears to disrupt the expression of sensory specific satiety may therefore result in a reduced susceptibility to the variety effect. This indicates that cafeteria diet fed rats may fail to “disinhibit” consummatory responses when given access to an assortment of novel, palatable foods. This is in contrast to literature describing the “buffet effect” whereby dietary variety promotes over consumption by switching to ingestion of novel foods (Rolls, 1981). Our data suggests that diets high in variety may override sensory specific satiety and promote consumption in general.

In the present experiments, rats fed the cafeteria diet consumed less of the palatable solutions than the chow fed rats. The reduced intake of palatable solutions is perhaps due to greater amounts of moisture in the cafeteria diet, thus the physiological impact of water restriction may be lessened, or to a lower hedonic value accruing to the solutions after constant exposure to a highly palatable diet in comparison to laboratory chow diet. Another alternative is that the decreased consumption in cafeteria diet fed rats was due to metabolic satiety, and that the decreased volumes consumed at test reflect this as opposed to impaired specific satiety. However, our analysis accounted for volume consumed during pre-exposure as a covariate factor, indicating that the expression of specific satiety was not influenced by the volume consumed. Furthermore, although we demonstrated that a limited pre-exposure volume of 10 ml was sufficient to evoke sensory specific satiety in chow fed rats, we did not test smaller volumes, as cafeteria diet rats consumed between 5–7 ml during pre-exposure. Additionally, following diet withdrawal ex-cafeteria diet fed rats consumed equal volumes overall of the solutions at test, yet exhibited a pronounced impairment in sensory-specific satiety, suggestive that this observation was not due to metabolic satiety.

These data suggest that cafeteria diet fed rats may fail to retain short-term information regarding recently consumed palatable foods (Henderson et al., 2013), and hence fail to exhibit sensory-specific satiety. Memory deficits and hippocampal dysfunction have been associated with diet-induced obesity (Greenwood and Winocur, 1990; Baybutt et al., 2002; Davidson et al., 2005; Granholm et al., 2008; Kanoski and Davidson, 2010, 2011; Darling et al., 2013), and these may contribute to overconsumption. In this model, a vicious cycle of obesity and deficits in hippocampal-dependent higher-order processes occur – including episodic memory (i.e., remembering what we have eaten) and our sensitivity to internal hunger and satiety cues (Davidson et al., 2007; Francis and Stevenson, 2011). This leads to impairments in inhibiting retrieval of the memory of the appetitive post-ingestive consequences of energy intake by environmental food-related cues, increasing the likelihood that those cues would evoke additional appetitive behavior (Davidson et al., 2005). However, it has been demonstrated hippocampal lesions do not influence sensory-specific satiety, or incentive value controlled instrumental responding in rats (Reichelt et al., 2011).

Habituation theory describe show sensory stimuli influence factors related to ingestive behavior, whereby responsiveness changes to foods and food-associated stimuli that are repeatedly experienced during a meal (Epstein et al., 1992, 2009; Raynor and Epstein, 2001). When people eat the same food during a meal they become habituated to the motivating properties of the food and decrease their consumption. Thus, when presented with a range of foods during meals the amount consumed increases, most likely because habituation is stimulus specific and because variety may introduce dishabituation effects (Raynor and Epstein, 2001). Exposure to the cafeteria diet which contains a variety of foods that are changed daily may have altered habituation to these foods and thus underpin the observed deficit in the expression of sensory-specific satiety.

Dopamine is proposed to play a role in motivated behaviors, and findings by Ahn and Phillips (1999) indicated that that NAc and PFC dopamine eﬄux may form an important signal encoding the relative incentive salience of foods and thus act as a determinant of the pattern of behaviors observed in sensory-specific satiety. Thus, our observation of impaired sensory-specific satiety in a rat model of dietary obesity may be a behavioral manifestation of mesocorticolimbic dopamine system dysfunction. The impact of diet-induced obesity may have effects on multiple brain regions, possibly impacting on levels of opioids (Woolley et al., 2007a,b) and/or dopaminergic transmission (Ahn and Phillips, 1999, 2002; Johnson and Kenny, 2010; Kenny et al., 2013).

## CONCLUSION

Our current findings demonstrate that exposure to obesogenic “cafeteria” diets disrupt both the expression of sensory-specific satiety and stimulus-outcome associations. These observations are of importance in the understanding of how obesity may impact upon the processing of appetitive outcomes and associated stimuli, and also to how maladaptive associations may control food seeking behavior in the absence of physiological and homeostatic requirements. Future studies should extend our current observations, further reducing the pre-exposure volume and interrogating the enduring nature of the sensory specific satiety deficit we observed following 1 week diet withdrawal, and also whether the cue-devaluation effect persists following diet withdrawal.

## Conflict of Interest Statement

The authors declare that the research was conducted in the absence of any commercial or financial relationships that could be construed as a potential conflict of interest.
